# Analysis of Recurrence Patterns Following Pterygium Surgery With Conjunctival Autografts

**DOI:** 10.1097/MD.0000000000000518

**Published:** 2015-01-30

**Authors:** Soo Hyun Kwon, Hong Kyun Kim

**Affiliations:** From the Department of Ophthalmology, Kyungpook National University School of Medicine, Daegu, South Korea.

## Abstract

The aim of this article is to evaluate and classify pathogenetic origins based on morphologic reproliferative patterns in patients who underwent pterygium excision with a conjunctival autograft.

In this retrospective, observational case series, a total of 116 eyes of 116 patients with pterygium who underwent pterygium excision with a conjunctival autograft between February 2009 and May 2011 were reviewed. Using consecutively recorded photographs, we evaluated preoperative morphologic severity, postoperative complications, recurrences, and growth patterns.

The regrowth of fibrovascular tissue was observed in 14 of our study cases (12.1%). Of these, 5 cases (4.3%) showed clinically significant recurrences. We observed 3 different morphologic patterns of recurrence: regrowth over the epithelial defect; transformation of the conjunctival graft into the pterygial tissue; and regrowth from unexcised pterygial tissue. Each recurrence pattern showed characteristic fibrovascular growth, the origin of this regrowth, and grade of severity. In 25 cases (21.6%), postoperative complications were observed. Of the analyzed variables, age <40 years (*P* = 0.019; odds ratio [OR], 5.82; 95% confidence interval [CI], 1.34–25.28) and the presence of postoperative complications (*P* = 0.008; OR, 6.32; 95% CI, 1.62–24.58) were statistically significant in multivariate analyses using logistic regression.

The use of conjunctival autografts for pterygium surgery is effective, but recurrences are observed in some cases exhibiting unique pathogenic patterns according to their origin. A complete understanding of the pathogenesis of these lesions based on their morphologic regrowth pattern will help to prevent recurrences in patients who undergo pterygium surgery.

## INTRODUCTION

Pterygium is considered a chronic lesion of the bulbar conjunctival tissue characterized by epithelial hyperplasia and elastotic degeneration.^1–8^ However, it also has invasive characteristics, including dysplastic expression, local invasiveness, and a high recurrence rate. The tumor-like characteristics of pterygium are expressed when reproliferation occurs after excisional surgery.^9,10^ Although various surgical procedures, including adjunctive treatments, have been proposed for the treatment of pterygium, recurrence remains a significant problem after surgical excision.^11–13^

In 1985, Kenyon et al^14^ first described the use of conjunctival autografts for the management of recurrent or advanced pterygium and reported a low recurrence rate of 5.3% with this method. Since this report, conjunctival autografts have been found to be a safe and effective option in pterygium surgery and have become the primary choice for the surgical management of this disorder.^15–17^ However, the procedure in question is technically demanding and the recurrence rates are variable, sometimes considerably, among different reports.^13,18,19^ Furthermore, there is no consensus regarding the conjunctival graft for use in pterygium surgery, including the necessity of limbal transplantation and the application of mitomycin C (MMC).^20–22^ Similarly, no comprehensive study regarding the pathogenetic origin of recurring pterygium after surgery is currently available.

In our present study, we performed a retrospective analysis of the morphologic changes that occur after pterygium excision and a conjunctival graft. Through this evaluation, we aimed to elucidate the pathogenetic origin of the reproliferative tissue. With respect to the origin of this tissue, we analyzed the risk factors and classified the patterns of recurrence.

## MATERIALS AND METHODS

We retrospectively reviewed the medical records of 116 eyes of 116 patients who underwent pterygium excision with conjunctival autograft for the treatment of pterygium between February 2009 and May 2011 at Kyungpook National University Hospital, Daegu, South Korea. The study protocol was approved by the Institutional Review Board of the Kyungpook National University Hospital. Written informed consent was obtained from all subjects after an explanation of the research purpose. This study was registered at ClinicalTrials.gov (identifier NCT02059837).

Patients who were indicated for surgery included those with cases of pterygium extending at least to the limbus and those with cases that showed prominent vascularization, thickened fibrotic proliferation resulting in difficulty observing the episcleral vessels, and a diplopia attributable to fibrous adhesion. Patients who had undergone follow-up examinations for at least 1 year after surgery were included in our study series. Patients with fibrovascular proliferation of the conjunctiva secondary to injury, a severe ocular surface disease such as blepharitis, severe dry eye syndrome, and systemic pathology that could affect wound healing after ocular surgery were excluded from the analysis. Written informed consent was obtained from all subjects after an explanation of the research purpose. The preoperative data collected included information on demographics, the number of previous pterygium excision surgeries, preoperative morphologic severity, and preoperative visual acuity.

Using the preoperative ocular examination, the examiners analyzed the morphologic severity of the pterygium and obtained images using the capturing system (Emedio, Seoul, Korea) of the slit-lamp biomicroscope. We reviewed the preoperative images of all patients and classified their morphologic severity based on our pterygium grading system. This grading system has been described in detail elsewhere^23^ and classifies the lesions on the basis of the pterygial relative thickness, increased vascularity, and the anatomical position of the abnormal fibrovascular head (Table 1). All surgical procedures were performed by the same single operator (H.K.K.). Lidocaine (2%) was injected into the subtenon for anesthesia, and the pterygial tissue and subconjunctival fibrovascular tissue were subsequently removed as broadly as possible. After complete and meticulous dissection of the head of the pterygium from the cornea, the body of the pterygium was dissected and excised using conjunctival scissors. Free conjunctival autografts were used in all patients. The conjunctival autograft was harvested from the superotemporal side of the same eye with a 1.0-mm margin of the limbal conjunctiva. In 55 eyes, a small surgical sponge soaked with 0.04% MMC solution was placed in contact with the upper and lower exposed scleral surfaces for 2 minutes during the surgery. Each graft was sutured using 10-0 nylon.

**TABLE 1:**
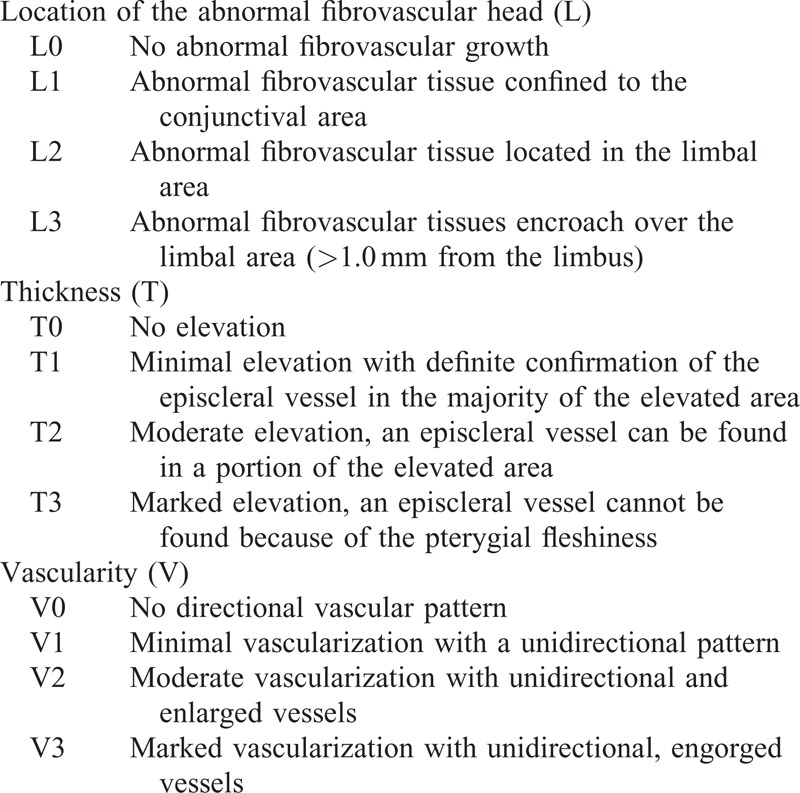
Standard Grading System of Pterygium

All patients received an identical regimen of topical agents: 1% prednisolone, 0.5% levofloxacin, and preservative-free 0.1% hyaluronic acid were applied 4 times daily for 2 weeks after the surgery. After 2 weeks, the 1% prednisolone acetate dosage was tapered to a twice-daily regimen for 2 weeks. The sutures were removed at 10 days after surgery. At 1 month after surgery, we began follow-up of the patients, which occurred every 2 months. Slit-lamp examinations were performed to check for recurrence at every visit, and the last image taken over the course of these examinations was used to determine whether the patient had experienced recurrence. Morphologic recurrence was defined as any fibrovascular proliferation from the original pterygium site and was classified using standardized methods. We defined clinically significant recurrence as pterygial regrowth that required a surgical procedure to remove proliferating conjunctival tissue growing over the cornea. Cases of symblepharous adhesion or pterygial grade of T3 or V3 were also included in the category of clinically significant recurrence. The postoperative data collected during the follow-up included postoperative visual acuity, early postoperative complications, recurrence, and other late postoperative complications.

Statistical analysis was performed by considering each risk factor for the recurrence and nonrecurrence groups. The analyzed parameters are indicated in Table 2. Fisher exact tests were used to analyze categorical variables such as gender and recurrence. *P* < 0.05 was considered significant. To evaluate the strengths of associations, logistic regression analysis was conducted and odds ratios (ORs) with 95% confidence intervals (CIs) were calculated. For multivariate data analysis, binary multiple logistic regression using backward conditional elimination was performed to identify significant independent factors associated with recurrence. SPSS version 19.0 (SPSS Inc, Chicago, IL) was used for these statistical analyses.

**TABLE 2:**
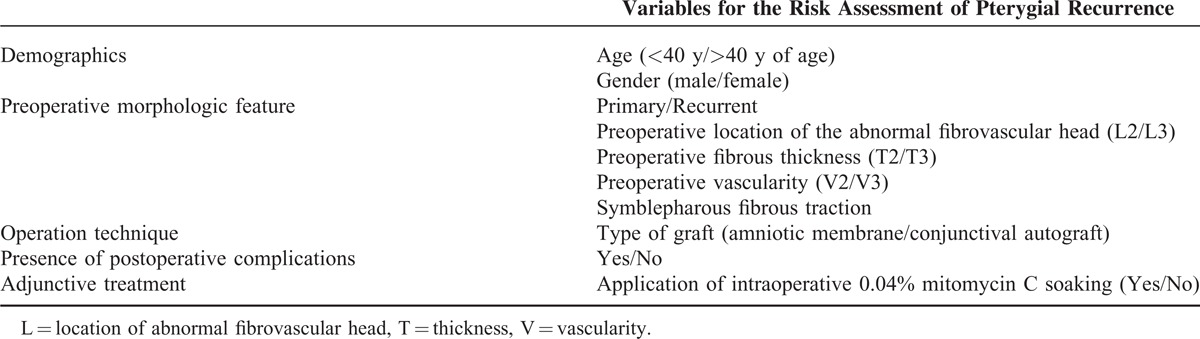
Analyzed Variables for Pterygium Recurrence

## RESULTS

A cohort of 116 eyes from 116 patients with primary or recurrent pterygium was reviewed in this study. Among these patients, 53 were men (45.7%) and 63 were women (54.3%). The mean age of the patients at the time of surgery was 56.2 ± 11.4 years (range, 24–82 years). The mean follow-up period was 25.8 ± 13.0 months (range, 12–72 months). Primary pterygium was found in 80 eyes (69.0%), whereas 36 eyes (31.0%) had undergone at least 1 previous pterygium surgery. Patient data and preoperative morphologic severities are listed in Table 3. The regrowth of fibrovascular tissue was observed in 14 cases (12.1%). Of these, 5 cases (4.3%) showed clinically significant recurrences. In the 14 cases showing recurrences, 3 distinct morphologic recurrence patterns were found. These were fibrovascular regrowth after epithelial defect of the graft in 3 cases, pterygial transformation of the graft in 5 cases, and regrowth from unexcised pterygial tissue in 6 cases. The clinical characteristics of 14 cases are summarized in Table 4.

**TABLE 3:**
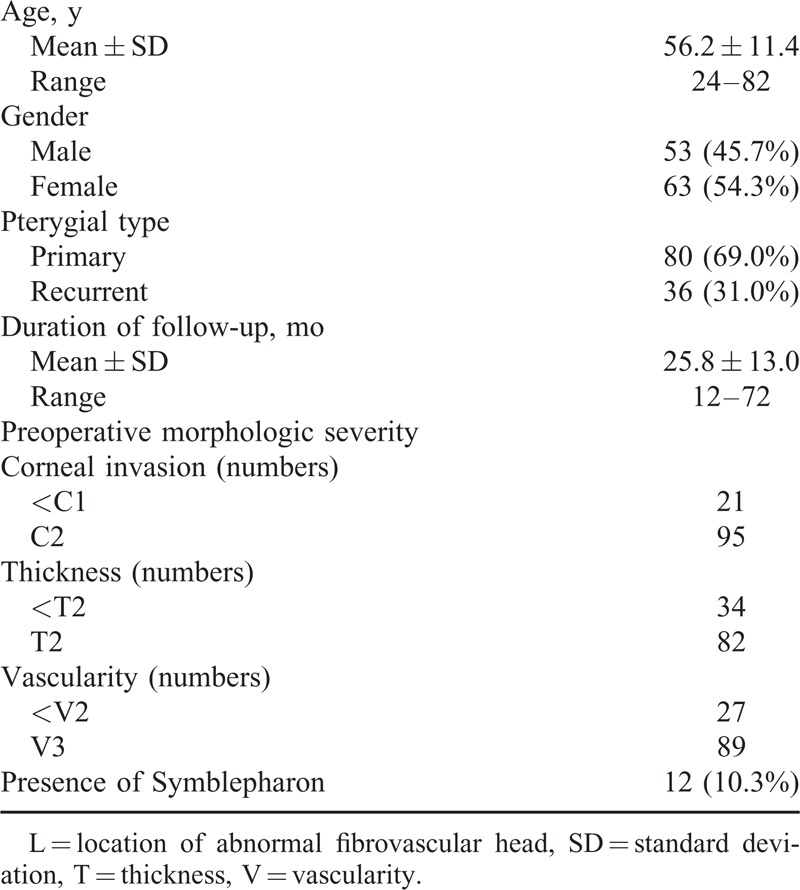
Patient Characteristics

**TABLE 4:**
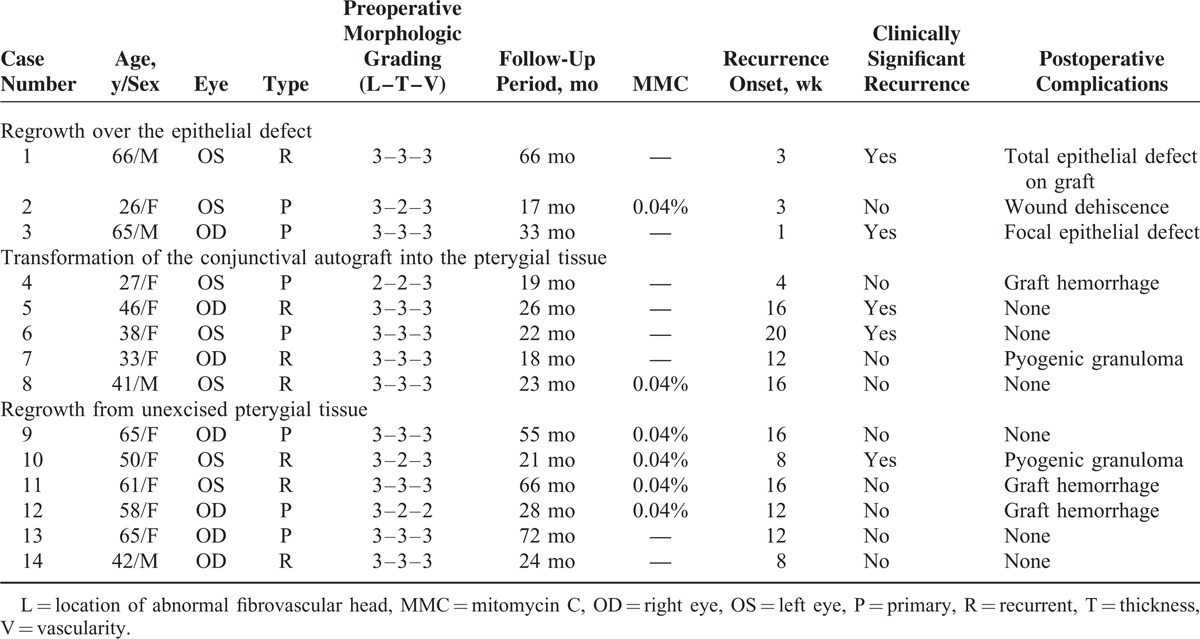
Representative Clinical Data for 14 Patients Among 116 Patients With Recurrent Pterygium Treated by Pterygium Excision With Conjunctival Autograft

### Regrowth Over the Epithelial Defect

**FIGURE 1:**
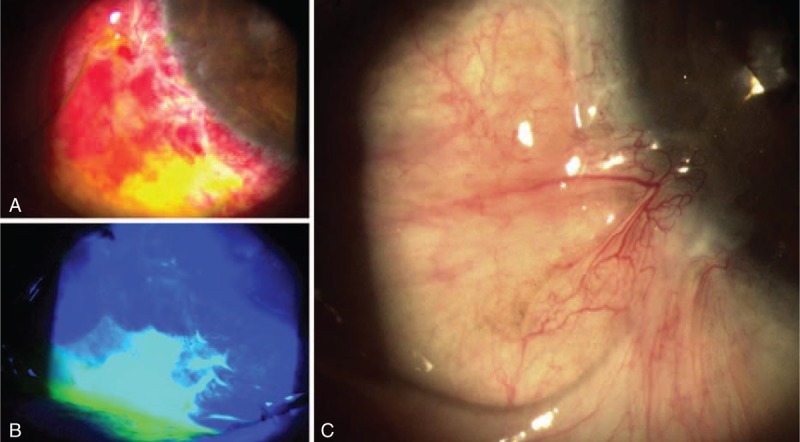
Postoperative slit-lamp photographs. (A) Slit-lamp and (B) fluorescein staining images on postoperative day 1; a clear margin of the epithelial defect of the conjunctiva is apparent. (C) Vigorous fibrovascular proliferation of the pterygial tissue is apparent over the previous defective epithelial area. The regrowth extended over the original site of the pterygium within 1 mo after surgery.

Regrowth over the epithelial defect was observed in 3 of our cases. Case 1 involved a 66-year-old man with bilaterally distributed primary pterygial heads on the left eye before surgery. The preoperative morphologic severity was L2–T2–V3 based on the standard pterygium grading system. A completely defective epithelium on the graft was observed at 1 day after surgery, although the graft was not lost. Aggressive fibrovascular growth was observed within 1 month. The reproliferative tissue extended over the original site of the pterygium. Case 2 involved a 26-year-old woman with wound dehiscence between the graft and recipient conjunctiva with a partial epithelial defect on the graft after surgery. The proliferating tissues over the site were accompanied by aggressive fibrovascular proliferation. Case 3 involved a 65-year-old man whose pterygial excision site showed an epithelial defect on the temporal side of the graft with aggressive regrowth of proliferative tissue over the defect (Figure 1).

### Transformation of the Autologous Conjunctival Graft Into the Pterygial Tissue

**FIGURE 2:**
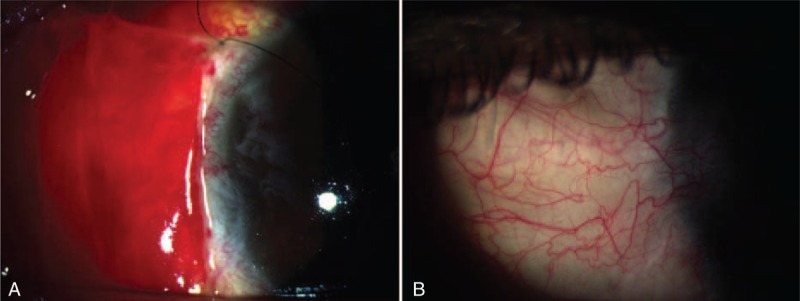
Postoperative slit-lamp photographs of case 4. (A) On postoperative day 7, subconjunctival hemorrhage is shown under the graft. (B) Regrowth of the pterygial tissue with newly formed vascularization observed at 3 mo postoperatively.

Transformation of the autologous conjunctival graft into the pterygial tissue originated from the grafted conjunctiva and was observed in 5 cases. Case 4 involved a 27-year-old woman who experienced recurrent pterygial excision and conjunctival autograft in the left eye. Graft hemorrhage was observed 2 weeks after the surgery. However, the hemorrhage was well absorbed and no other significant sign of recurrence was observed during the early postoperative period. Fibrous proliferation started from the graft after 1 month, and vasculature from the graft followed thereafter. This type of recurrence was characterized by a very thin and mild growth pattern. The reproliferating tissue did not grow over the previous pterygial head margin (Figure 2).

### Regrowth From Unexcised Pterygial Tissue

**FIGURE 3:**
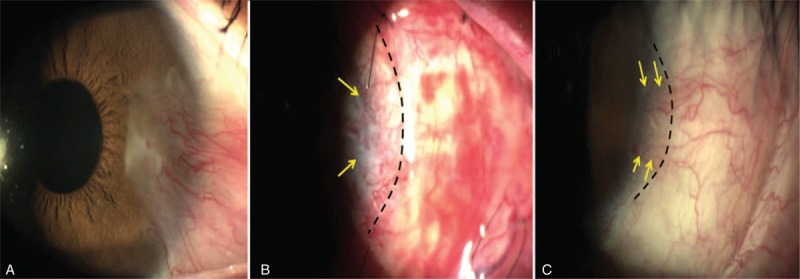
(A) Preoperative slit-lamp photograph of case 14. (B) Postoperative day 6. Dotted line indicates the limbal margin of graft. Tightly adherent unexcised pterygial tissue on the bed can be observed (arrows). (C) Slit-lamp photograph at 8 wk postoperatively. Arrows indicate the recurrent fibrovascular tissue originating from the tightly adherent unexcised pterygial bed and dotted line indicates the limbal margin of the graft.

Regrowth of the remaining pterygial tissue was another pattern of recurrence, and this was observed in 6 cases. The remaining tissue originated from 3 sources: the remnant pterygial tissue around the superior or inferior border of the excision; the nasal margin of the excised pterygial tissue; and the tightly adherent pterygial tissue on the excised bed. Outflanking from the remnant pterygial tissue around the superior or inferior margin was observed in 4 cases, including case 11. Case 12 was an example of growth from the nasal remnant pterygial tissue. Fibrous proliferation originated from the tightly adherent unexcised pterygial bed in case 14 (Figure 3).

### Postoperative Complications

Postsurgical complications were observed in 25 cases. Pyogenic granuloma on the donor site was noted in 9 cases and on the graft site in 6 cases. There were 5 cases of graft hemorrhage, 4 cases of inclusion cyst, 1 case of wound dehiscence with epithelial defect between the graft and recipient conjunctiva, and 2 cases of epithelial defect on donor graft. Of the 14 cases in which recurrence was noted, 8 cases showed a postoperative complication. Recurrent proliferation was observed in 3 cases of 5 graft hemorrhage cases. Of the 6 cases of pyogenic granuloma on the graft site, 2 showed recurrences. All 3 cases of defective postoperative graft epithelium showed aggressive recurrences.

### Recurrence Risk Factors

We examined the risk factors for recurrence in our study series. Age, but not gender, was found to be a statistically significant factor for recurrence. However, the preoperative morphologic severity was not associated with recurrence. The incidence of recurrence was higher in patients who had undergone a previous pterygium surgery compared with the primary pterygium group, but this difference was not statistically significant. During the operations for our patients, we used a 0.04% MMC soaked for 2 minutes as an adjunctive treatment in selected cases. However, the use of MMC did not lower the incidence of recurrence. Of the analyzed variables, age <40 years (*P* = 0.019; OR, 5.82; 95% CI, 1.34–25.28) and the presence of a postoperative complication (*P* = 0.008; OR, 6.32; 95% CI, 1.62–24.58) were found to be statistically significant in multivariate analysis using a logistic regression model.

## DISCUSSION

Since Kenyon et al^14^ first described their conjunctival autograft procedure for the management of pterygium, this surgical technique has been considered the most effective and safest procedure for reducing recurrence while avoiding the risk of potentially serious complications.^11,15–17,24,25^ However, some studies of conjunctival autografts to treat pterygium have still shown a significant rate of recurrence.^13,18,19^ No study has yet defined the pathogenetic origin of reproliferation. To lower the incidence of recurrence, it is necessary to understand the origin of recurrence. We observed morphologic regrowth after surgery in 14 of our study cases (12.1%), with 5 cases (4.31%) showing a clinically significant recurrence. These findings are similar to those of a previous report.^11,26^ In our current retrospective series, we defined recurrence as any degree of fibrovascular invasion on the normal ocular surface, including the area of the conjunctival graft. All proliferation was taken into consideration in our morphologic analysis of the recurrence pattern rather than just clinical concerns. Thus, minimal proliferation was included in our recurrence group.

Two recurrence patterns have been previously reported after conjunctival autografts: cross-graft recurrence and outflanking.^16^ Other authors have proposed that recurrence was caused only by reproliferation of previously unexcised pterygial tissue. However, we observed 2 other mechanisms of recurrence. In our current study, we identified 3 types of recurrence patterns. In the first type, compromised conjunctival epithelial integrity over the entire excised area of the pterygial tissue induced vigorous fibrovascular proliferation. An epithelial defect of any origin can induce this proliferation. This occurrence has been frequently observed with the bare sclera method.^27–29^ To cover the defective ocular surface, epithelialization is initiated from the surrounding tissue, followed by significant fibrovascular proliferation. This recurrence pattern showed the most aggressive proliferation of the 3 patterns we observed, with proliferation usually occurring over the graft. The observed proliferation was more aggressive as the size of the defective epithelial area increased. A total epithelial defect was observed in 1 of our cases. This mechanism of recurrence is relatively aggressive and thick. To prevent this recurrence pattern, grafting of the intact and healthy conjunctival epithelium should occur. We assumed that the healthy epithelial graft would act as a tight barrier against abnormal fibrovascular proliferation and prevent the triggering of an aggressive epithelial regenerative signal. The causes of the defect were wound dehiscence, primary graft failure, and a focally defective epithelial graft. A small graft or excessive excision of the surrounding conjunctiva can cause dehiscence. The second cause of a defective conjunctival epithelium is whole or focal graft damage during the operation. An extended operation period and surgical damage of the autograft attributable to a lack of skill or inexperience increase damage to the conjunctival epithelium and can affect the incidence of recurrence.^7^ Dryness or irrigating solution can be toxic to the conjunctival epithelium. Therefore, an intact and healthy conjunctival epithelium is essential for avoiding this serious pattern of recurrence.

The transformation of the epithelial graft into the pterygial tissue is another pathogenic mechanism of proliferation. The factors related to this transformation are not yet fully understood. By examining serial photographs of our recurrent cases herein, we concluded that the origin of this recurrence is the grafted conjunctiva. This type of recurrence showed mild thickness and very slow progression. The surface haziness started from the graft, and the observed out-budding vasculature from the graft toward the hazed surface did not proliferate over its previously involved margin. This type of recurrence showed slight similarity to the growth pattern of the primary pterygial fibrovascular growth. Although the exact mechanism was unclear in our morphologic analyses, we presumed that this type of proliferation was related to the primary pterygium, such as ultraviolet light damage.

The third mechanism of pterygium recurrence is regrowth of the remnant pterygial tissue. This pattern was recognized in a previous report.^16^ The tissue can originate from the body of the pterygium or the remnant tissue at the limbal area. In cases of recurrence attributable to remnant pterygial tissue, the tissue usually adheres tightly to the surface and it cannot be easily removed. Although the incidence of recurrence between primary and recurrent pterygium was not found to be different, this mechanism of recurrence occurred in the recurrent pterygium. In contrast to the proliferation of the epithelial defect, this proliferation was less vigorous and grew slowly, which might have been attributable to an intact healthy epithelial barrier inhibiting aggressive growth. Thus, to reduce recurrence, it is important to have clear and complete excision.

In our present study, age <40 years and the presence of postoperative complications were statistically significant risk factors for recurrence. Although the exact mechanism is not yet clear, the wound healing process may be affected by the patient's age. On the basis of our present observations, the timing of the surgery is very important for the treatment of a primary pterygium, which typically has a slow progressive nature. Although postoperative complications were limited and mild in our cases, they were found to be related to recurrence. Wound dehiscence, pyogenic granuloma, and graft hemorrhage will cause an epithelial defect, and the resulting uncontrolled inflammation will be related to the triggering of recurrent growth. Graft edema was observed in almost all of our current cases during the early postoperative period. For stable grafting, mechanical rubbing and inflammation of the graft should be avoided during this period.

Although our morphologic evaluations after pterygium excision and conjunctival autograft elucidated the cause of recurrence, we could not fully evaluate the underlying mechanisms. The retrospective nature of our current study may have resulted in a lack of statistical power. For a better understanding of recurrence after pterygium surgery, a prospective randomized study that includes histological examination is necessary in the future. However, with our current findings, we are able to propose a clinical strategy for reducing the incidence of recurrence after pterygial excision and conjunctival autograft based on the elucidation of the regrowth pattern of the pterygium. Early surgery is not recommended for young patients with pterygium, and surgical indication should be adjusted in accordance with the patient's age. Regrowth from the epithelial defect was the most aggressive we observed and induced serious fibrovascular proliferation. To avoid this type of recurrence, an intact conjunctival epithelial graft is essential. Thus, a careful approach should be taken with the surgical technique and postoperative care should be used to treat these cases.

In conclusion, a conjunctival autograft is a very safe and effective surgical approach to the treatment of pterygium, and morphologic evaluations with standardization for recurrent patterns of pterygium should facilitate an understanding of the mechanisms of reproliferation and enable the prevention of serious recurrence patterns.
